# Supplementary material for: Recognition of Community Pharmacists’ Behaviors Related to Information Sharing: A Cross-Sectional Study
Source: Pharmacy (Basel). 2024 Apr 6;12(2):63. doi: 10.3390/pharmacy12020063 (PMC11053978; doi:10.3390/pharmacy12020063)
Supplement: Supplementary file 1 [file pharmacy-12-00063-s001.zip › supplementary_materials_1.pdf]

Supplementary Materials

Survey contents

1) Common items for both pharmacists and patients

Pharmacists' behaviors related to information sharing

1	Talk with patients about how to watch for medication side effects.
2	Talk with patients even if the patients don't have any medication questions.
3	Talk with patients about whether or not it is OK to take their medications with over-the-counter products.
4	Show an interest in working with patients to meet their healthcare needs.
5	Communicate a desire to help patients manage their medications.
6	Make sure that patients understand how to use their medications before they leave the pharmacy
7	Communicate a desire to help patients with their medication concerns.
8	Listen to patients when they have a medication question.
9	Be easily approachable to discuss a patient's medication concerns.

Seven-point Likert scale, from 1, "very strongly disagree," to 7, "very strongly agree."

2) Items for patients

Trust in community pharmacist

1	I feel I can consult the pharmacist at all times.
2	I feel at ease when the pharmacist listens to what I have to say.
3	I feel relaxed after speaking to the pharmacist.
4	I feel supported by the pharmacist when I have questions or concerns about medications for treatment.
5	When I have questions about medication, I want to consult the pharmacist.
6	I think that the pharmacist understands my perspective.
7	I feel that the pharmacist can be trusted to keep my secrets.

Six-point Likert scale, from 1, “very strongly disagree,” to 6, “very strongly agree.”

Patient willingness to self-disclose to the community pharmacist

1	I would actively inform the pharmacist about my history of illness(es) for which I have been taking medications from the pharmacy(ies).
2	I would actively inform the pharmacist about the explanations provided to me by my doctor regarding treatment using the drugs.
3	I would actively speak to the pharmacist about my questions and concerns about drug treatments.
4	I would actively speak to the pharmacist if I took a drug prescribed by a doctor and experienced unusual symptoms or any changes in my physical condition.
5	I would actively inform the pharmacist of the results of blood tests performed at hospitals.

Seven-point Likert scale, from 1, “very strongly disagree,” to 7, “very strongly agree.”

Patient characteristics

Age
Gender
Number of medications they took
Number of pharmacies they went to regularly
An explanation was provided by the family pharmacist
Medical conditions them (self-report)
Hypertension
Heart diseases (e.g., angina pectoris, myocardial infarction, and arrhythmia)
Diabetes mellitus
Dyslipidemia
Gout and hyperuricemia
Osteoporosis
Allergic disease
Respiratory diseases (including asthma)
Migraine headache
Back pain and hernia
Rheumatoid arthritis
Diseases related to the thyroid gland
Diseases of digestive organs (stomach, duodenum, and small and large intestines)
Skin diseases
Hepatic diseases
Renal diseases
Gynecological diseases
Urological diseases
Psychiatric diseases
Insomnia
Cancer

3) Items for pharmacists

Pharmacist characteristics

Years of experience
Gender
Certifications
Education system-qualified by the Japan Education Pharmacists Center
Primary care-certified pharmacist
Board-certified pharmacist in home care pharmacy
JPEC-certified pharmacist in pediatric pharmacotherapy
Pharmacist certified in Chinese and other herbal medicines
Certified practical training supervisor pharmacist
