# Supplementary material for: Recognition of Community Pharmacists’ Behaviors Related to Information Sharing: A Cross-Sectional Study
Source: Pharmacy (Basel). 2024 Apr 6;12(2):63. doi: 10.3390/pharmacy12020063 (PMC11053978; doi:10.3390/pharmacy12020063)
Supplement: Supplementary file 1 [file pharmacy-12-00063-s001.zip › supplementary_materials_2.pdf]

Supplementary Materials 2

Questionnaire for pharmacists

We recently launched a "Survey of Pharmacists' Recognition of Patient Interaction". The survey is open to pharmacists who have worked in community pharmacies for at least one year. The survey is unregistered. We will not use your responses for any purpose other than this study, nor will we disclose them to any outside party or to the detriment of any individual. All information will remain strictly confidential. If you feel that you no longer wish to cooperate with us in the middle of the survey, you may do so. Your candid responses will be greatly appreciated.

The research is supported by JSPS KAKENHI (grant number JP17K09244). After approval by the Ethics Committee for Research Involving Human Subjects of the Showa University Graduate School of Pharmaceutical Sciences, the research will be conducted with the approval of the Dean of the Graduate School of Pharmaceutical Sciences. The research will be presented at a conference and published in a peer-reviewed journal, and the response data will be kept for three years after the presentation and then destroyed. If you would like to review the research protocol or have any other questions, please contact the following person.

Thank you for your cooperation.

Principal Researcher

Keiko Kishimoto, Department of Social Pharmacy, Showa University Graduate School of Pharmacy

1-5-8, Hatanodai, Shinagawa-ku, Tokyo, 142-8555

Tel +813-3784-8016

kishimoto-k@pharm.showa-u.ac.jp

Q0. If you have read the instructions above and agree that your responses may be used for research purposes, please check the box below and respond. [☐ agree, ☐ disagree]

Q1. How long have you worked in an insurance pharmacy? Please choose one that applies. [1 = less than 1 year, 2 = more than 1 year]

Q2. Please provide your interaction with the patient. [1 = completely disagree, 2 = disagree, 3 = tend to disagree, 4 = neither, 5 = tend to agree, 6 = agree, 7 = strongly agree].

2-1. Talk with patients about how to watch for medication side effects

2-2. Talk with patients even if the patients don't have any medication questions

2-3. Talk with patients about whether or not it is OK to take their medications with over-the-counter products

2-4. Show an interest in working with patients to meet their healthcare needs

2-5. Communicate a desire to help patients manage their medications

2-6. Make sure that patients understand how to use their medications before they leave the pharmacy

2-7. Communicate a desire to help patients with their medication concerns

2-8. Listen to patients when they have a medication question

2-9. Be easily approachable to discuss a patient's medication concerns

Q3. Please provide how many years of experience you currently have working as a pharmacist.

Q4. Please tell us your gender. [1 = male, 2 = female]

Q5. Please select all certifications that apply to you as a qualified pharmacist.

5-1. Education system-qualified by the Japan Education Pharmacists Center

5-2. Primary care-certified pharmacist

5-3. Board-certified pharmacist in-home care pharmacy

5-4. JPEC-certified pharmacist in pediatric pharmacotherapy

5-5. Pharmacist certified in Chinese and other herbal medicines

5-6. Certified practical training supervisor pharmacist

Questionnaire for pharmacists (in Japanese)

このたび、我々は「薬局薬剤師の患者応対の認識に関する調査」に着手しました。これに伴い、保険薬局勤務経験が 1 年以上の薬剤師を対象に調査を行うこととなりました。このアンケートは無記名で行います。回答頂いた内容を本研究以外に用いたり、外部に漏らしたり、個人の不利益につながるようことは致しません。すべての情報は厳密な保護を行います。回答への協力を止めたいと途中で感じた場合、中断いただいても問題ありません。率直な回答を宜しくお願い致します。

本研究は、文部科学省 科研費（基盤 C 17K09244）より実施するものです。また、昭和大学薬学研究科人を対象とする研究等に関する倫理委員会の承認後、薬学研究科長の研究実施許可を得て実施しております。研究成果については学会での発表及び学術雑誌への論文掲載を予定しており、発表から 3 年間、回答データを保存し、その後、廃棄します。

研究計画書の閲覧やその他お問い合わせたいことがございましたら、下記研究責任者までご連絡ください。

ご協力を賜りますよう、どうぞ宜しくお願い申し上げます。

研究責任者

昭和大学薬学部　社会健康薬学講座社会薬学部門　岸本桂子

〒142-8555 東京都品川区旗の台 1-5-8 Tel 03-3784-8016

kishimoto-k@pharm.showa-u.ac.jp

Q0. 上記の説明をお読みになり、ご回答いただいた内容が研究目的で使用されることに同意される方は、以下のチェックボックスにチェックを入れてご回答ください。[□ 同意する、□ 同意しない]

Q1. 保険薬局での勤務経験はどのくらいですか。あてはまる方を 1 つ選んでください。[1=1 年未満, 2=1 年以上]

Q2. あなたと患者のやりとりについて教えてください。[1=全くあてはまらない, 2=ほとんどあてはまらない, 3=あまりあてはまらない, 4=どちらとも言えない, 5=少しあてはまる, 6=ほとんどあてはまる, 7=非常にあてはまる]

2-1. 薬の副作用をどう観察するかについて、患者と対話する

2-2. 患者から薬の質問が無い場合でも、患者と対話する

2-3. OTC 薬を併用してもよいかについて、患者と対話する

2-4. 患者の健康の維持増進に関する希望を叶えるために、患者をサポートする意思があることを示す

2-5. 薬の管理の支援をしたいと患者に伝える

2-6. 患者が薬局を去る前に、薬の使い方を理解しているかを確認する

2-7. 薬の心配事に対する支援をしたいと患者に伝える

2-8. 患者が薬に対する疑問を抱いているとき、患者の話を聞く

2-9. 薬に関する心配事について、患者が気軽に話しやすい雰囲気である

Q3. あなたは、現在、薬剤師として勤務経験何年目か教えて下さい。

Q4. あなたの性別を教えてください。[1=男性, 2=女性]

Q5. あなたが取得している認定薬剤師の資格として、あてはまるものを全てお選びください。

5-1. 研修認定薬剤師

5-2. プライマリ・ケア認定薬剤師

5-3. 在宅療養支援認定薬剤師

5-4. 小児薬物療法認定薬剤師

5-5. 漢方・生薬認定薬剤師

5-6. 認定実務実務実習指導薬剤師
