# Supplementary material for: Recognition of Community Pharmacists’ Behaviors Related to Information Sharing: A Cross-Sectional Study
Source: Pharmacy (Basel). 2024 Apr 6;12(2):63. doi: 10.3390/pharmacy12020063 (PMC11053978; doi:10.3390/pharmacy12020063)
Supplement: Supplementary file 1 [file pharmacy-12-00063-s001.zip › supplementary_materials_3.pdf]

Supplementary Material 3

Questionnaire for patients

We recently launched a "Survey of Pharmacists' Recognition of Patient Interaction". The survey is open to patients between the ages of 20 and 89. The survey is unregistered. We will not use your responses for any purpose other than this study, nor will we disclose them to any outside party or to the detriment of any individual. All information will remain strictly confidential. If you feel that you no longer wish to cooperate with us in the middle of the survey, you may do so. Your candid responses will be greatly appreciated.

The research is supported by JSPS KAKENHI (grant number JP17K09244). After approval by the Ethics Committee for Research Involving Human Subjects of the Showa University Graduate School of Pharmaceutical Sciences, the research will be conducted with the approval of the Dean of the Graduate School of Pharmaceutical Sciences. The research will be presented at a conference and published in a peer-reviewed journal, and the response data will be kept for three years after the presentation and then destroyed. If you would like to review the research protocol or have any other questions, please contact the following person.

Thank you for your cooperation.

Principal Researcher

Keiko Kishimoto, Department of Social Pharmacy, Showa University Graduate School of Pharmacy

1-5-8, Hatanodai, Shinagawa-ku, Tokyo, 142-8555

Tel +813-3784-8016

kishimoto-k@pharm.showa-u.ac.jp

Q0. If you have read the instructions above and agree that your responses may be used for research purposes, please check the box below and respond. [☐ agree, ☐ disagree]

Q1. Please provide your impression of the pharmacist [1 = completely disagree, 2 = disagree, 3 = tend to disagree, 4 = agree to agree, 5 = agree, 6 = strongly agree].

1-1. I feel I can consult the pharmacist at all times

1-2. I feel at ease when the pharmacist listens to what I have to say

1-3. I feel relaxed after speaking to the pharmacist

1-4. I feel supported by the pharmacist when I have questions or concerns about medications for treatment

1-5. When I have questions about medication, I want to consult the pharmacist

1-6. I think that the pharmacist understands my perspective

1-7. I feel that the pharmacist can be trusted to keep my secrets

Q2. Please answer these questions by assuming that you are in the following situations [1 = completely disagree, 2 = disagree, 3 = tend to disagree, 4 = neither, 5 = tend to agree, 6 = agree, 7 = strongly agree].

2-1. I would actively inform the pharmacist about my history of illness(es) for

which I have been taking medications from the pharmacy(ies).

2-2. I would actively inform the pharmacist about the explanations provided to me by my doctor regarding treatment using the drugs.

2-3. I would actively speak to the pharmacist about my questions and concerns about drug treatments.

2-4. I would actively speak to the pharmacist if I took a drug prescribed by a doctor and experienced unusual symptoms or any changes in my physical condition.

2-5. I would actively inform the pharmacist of the results of blood tests performed at hospitals.

Q3. Please provide your interaction with the community pharmacist. [1 = completely disagree, 2 = disagree, 3 = tend to disagree, 4 = neither, 5 = tend to agree, 6 = agree, 7 = strongly agree].

3-1. The community pharmacists talk with you about how to watch for medication side effects

3-2. The community pharmacists talk with you even if you don’t have any medication questions

3-3. The community pharmacists talk with you about whether or not it is OK to take your medications with over-the-counter products

3-4. The community pharmacists show an interest in working with you to meet your healthcare needs

3-5. The community pharmacists communicate a desire to help you manage your medicines

3-6. The community pharmacists makessure that you understand how to use your medications before you leave the pharmacy

3-7. The community pharmacists communicate a desire to help you with your medication concerns

3-8. The community pharmacists listen to you when you have a medication question

3-9. The community pharmacists are easily approachable to discuss your medication concerns

Q4. Please tell us your age (years old)

Q5. Please tell us your gender. [1 = male, 2 = female]

Q6. How many types of medicines are you prescribed by a doctor during regular hospital visits (please describe)?

Q7. How many pharmacies do you visit for your prescriptions regularly (please describe)?

Q8. Do you get an explanation from your "family pharmacist" when you pick up your medications at the pharmacy? [1=Yes, 2=No]

Q9. For which of the following diseases (excluding diseases in the fields of dentistry and ophthalmology) do you regularly visit a hospital/clinic for and receive medication prescriptions? Please select all that apply.

9-1. Hypertension

9-2. Heart diseases (e.g. angina pectoris, myocardial infarction, and arrhythmia)

9-3. Diabetes mellitus

9-4. Dyslipidemia (e.g. hyperlipidemia and hypercholesterolemia)

9-5. Gout and hyperuricemia

9-6. Osteoporosis

9-7. Allergic disease

9-8. Respiratory diseases (including asthma)

9-9. Migraine headache

9-10. Back pain and hernia

9-11. Rheumatoid arthritis

9-12. Diseases related to the thyroid gland

9-13. Diseases of digestive organs
(stomach, duodenum, and small and large intestines)

9-14. Skin diseases

9-15. Hepatic diseases

9-16. Renal diseases

9-17. Gynecological diseases

9-18. Urological diseases

9-19. Psychiatric diseases

9-20. Insomnia

9-21. Cancer

Questionnaire for patients (in Japanese)

このたび、我々は「薬局薬剤師の患者応対の認識に関する調査」に着手しました。これに伴い、20~89歳の患者を対象に調査を行うこととなりました。このアンケートは無記名で行います。回答頂いた内容を本研究以外に用いたり、外部に漏らしたり、個人の不利益につながるようことは致しません。すべての情報は厳密な保護を行います。回答への協力を止めたいと途中で感じた場合、中断いただいても問題ありません。率直な回答を宜しくお願い致します。

本研究は、文部科学省 科研費（基盤C 17K09244）より実施するものです。また、昭和大学薬学研究科人を対象とする研究等に関する倫理委員会の承認後、薬学研究科長の研究実施許可を得て実施しております。研究成果については学会での発表及び学術雑誌への論文掲載を予定しており、発表から3年間、回答データを保存し、その後、廃棄します。

研究計画書の閲覧やその他お問い合わせたいことがございましたら、下記研究責任者までご連絡ください。

ご協力を賜りますよう、どうぞ宜しくお願い申し上げます。

研究責任者

昭和大学薬学部　社会健康薬学講座社会薬学部門　岸本桂子

〒142-8555 東京都品川区旗の台 1-5-8 Tel 03-3784-8016

kishimoto-k@pharm.showa-u.ac.jp

Q0. 上記の説明をお読みになり、ご回答いただいた内容が研究目的で使用されることに同意される方は、以下のチェックボックスにチェックを入れてご回答ください。[□ 同意する、□ 同意しない]

Q1. 薬局の薬剤師に対するあなたの印象をお答えください。[1=全くそう思わない, 2=そう思わない, 3=どちらかといえばそう思わない, 4=どちらかといえばそう思う, 5=そう思う, 6=非常にそう思う]

1-1. 薬局の薬剤師には、いつも相談できると感じる

1-2. 薬局の薬剤師に話を聞いてもらうと、安心する

1-3. 薬局の薬剤師に相談すると、気持ちが楽になると思う

1-4. 薬による治療について悩んでいるとき、薬局の薬剤師は私を支えてくれるだろうと感じる

1-5. 薬による治療のことがわからないときは、薬局の薬剤師に、相談してみようという気になる

1-6. 薬局の薬剤師は、私の立場で気持ちを理解してくれていると思う

1-7. 薬局の薬剤師は、私の秘密を守ってくれると思う

Q2. 以下のような場面を仮定して回答してください。[1=全くあてはまらない, 2=ほとんどあてはまらない, 3=あまりあてはまらない, 4=どちらとも言えない, 5=少しあてはまる, 6=ほとんどあてはまる, 7=非常によくあてはまる]

2-1. 薬局で受け取っている薬の病気のこれまでの経緯を、薬局の薬剤師に積極的に伝える。

2-2. 薬による治療に関する医師からの説明内容を、薬局の薬剤師に積極的に伝える。

2-3. 薬に関する不安や疑問を、薬局の薬剤師に積極的に相談する。

2-4. 医師から処方されている薬を飲んで、いつもと違う症状、体調の変化がでたら、薬局の薬剤師に積極的に相談する。

2-5. 病院で行った血液検査の結果を、薬局の薬剤師に積極的に提示する。

Q3. あなたと薬局薬剤師とのやりとりについて教えてください。[1=全くあてはまらない, 2=ほとんどあてはまらない, 3=あまりあてはまらない, 4=どちらとも言えない, 5=少しあてはまる, 6=ほとんどあてはまる, 7=非常によくあてはまる]

3-1. 薬局の薬剤師は、薬の副作用をどう観察するかについて、あなたと対話する

3-2. 薬局の薬剤師は、あなたに薬の質問が無い場合でも、あなたと対話する

3-3. 薬局の薬剤師は、市販薬を併用してもよいかについて、あなたと対話する

3-4. 薬局の薬剤師は、あなたの健康の維持増進に関する希望を叶えるためにサポートする意思があることを示す

3-5. 薬局の薬剤師は、あなたの薬の管理を支援したいと、あなたに伝える

3-6. 薬局の薬剤師は、あなたが薬局を出る前に、薬の使い方を理解しているか確認する

3-7. 薬局の薬剤師は、あなたの薬に関する心配事を支援したいと、あなたに伝える

3-8. 薬局の薬剤師は、あなたが薬に対する疑問を抱いているとき、話を聞く

3-9. 薬局の薬剤師は、あなたが薬に関する心配事について、気軽に話しやすい雰囲気である

Q4. あなたの年齢を教えてください。

Q5. あなたの性別を教えてください。[1=男性, 2=女性]

Q6. あなたが定期的に病院を受診して、医師から処方されている薬は全部で何種類ですか？

Q7. 処方薬を受け取るために定期的に通っている薬局は何か所ですか？

Q8. 薬局で薬を受け取る際、「かかりつけ薬剤師」から説明を受けていますか？
[1=Yes, 2=No]

Q9. あなたが病院・クリニックに定期的に受診し、薬の処方を受けている病気はどれですか（歯科、眼科は除く）。該当するもの全てを選択してください。

9-1. 高血圧

9-2. 心臓の病気（狭心症、心筋梗塞、不整脈等）

9-3. 糖尿病

9-4. 脂質異常症（高脂血症、高コレステロール血症）

9-5. 痛風、高尿酸血症

9-6. 骨粗しょう症

9-7. アレルギー・花粉症

9-8. 呼吸器の病気（喘息含む）

9-9. 片頭痛

9-10. 腰痛、ヘルニア

9-11. 関節リウマチ

9-12. 甲状腺に関する病気

9-13. 消化器（胃・十二指腸、小腸、大腸）の病気

9-14. 皮膚の病気

9-15. 肝臓の病気

9-16. 腎臓の病気

9-17. 婦人科の病気

9-18. 泌尿器の病気

9-19. 精神科の病気

9-20. 不眠症

9-21. がん
